# Understanding the associations between the number of close friends and life satisfaction: Considering age differences

**DOI:** 10.3389/fpsyg.2023.1105771

**Published:** 2023-03-28

**Authors:** Weixi Kang

**Affiliations:** Department of Brain Sciences, Imperial College London, London, United Kingdom

**Keywords:** friendship, life satisfaction, age, socioemotional selectivity theory, social support

## Abstract

Life satisfaction refers to one's subjective evaluation of life, which is the cognitive aspect of subjective well-being. Understanding factors that contribute to life satisfaction has important implications as higher life satisfaction is closely associated with better physical, psychological, and behavioral health outcomes. Close friendship serves as a valuable source of social support across life spans. Although there are some studies regarding the associations between friendship and well-being, much less is known regarding the relationships between the number of close friends and life satisfaction and how this association varies with age. By analyzing data from 29,785 participants with an age range of 16–101 years old from the Understanding Society, the current study found that there is a significant interaction effect of age with the number of close friends (*b* = −0.003, *p* < 0.01, 95% C.I. [−0.004, −0.001]) after controlling for demographic covariates. Simple slope regressions showed that the positive association between the number of close friends and life satisfaction is the strongest in young people (*b* = 0.018, *p* < 0.001, 95% C.I. [0.012, 0.024]), and less strong in middle-aged (*b* = 0.008, *p* < 0.001, 95% C.I. [0.003, 0.013]), and the weakest in older adults (*b* = 0.004, *p* < 0.01, 95% C.I. [0.002, 0.007]).

## Introduction

Life satisfaction refers to one's subjective evaluation of life, which is the cognitive aspect of subjective well-being. Understanding factors that contribute to life satisfaction has important implications as higher life satisfaction is closely associated with better physical, psychological, and behavioral health outcomes such as a reduced risk of mortality (Koivumaa-Honkanen et al., 2000), depression (Koivumaa-Honkanen et al., 2004), physical functioning limitations (Kim et al., 2021), chronic pain (Dezutter et al., 2010), sleep problems (Ness and Saksvik-Lehouillier, 2018), negative affect (Singh and Jha, 2008), perceived constraints (Kim et al., 2021), and loneliness (Ozben, 2013); but also with increased physical activities (Zullig and White, 2011), positive affect (Singh and Jha, 2008), and optimism (Ho et al., 2010). All of these health outcomes are closely related to the healthcare system in society. Several theoretical models have been developed to investigate the contributing factors of life satisfaction, which include a bottom-up, top-down, and integrated account (e.g., Lachmann et al., 2017; Malvaso and Kang, 2022). Recently, the evidence seems to favor the integrated account of life satisfaction, which posits that life satisfaction is made up of demographics (e.g., age), satisfaction with aspects of life (e.g., satisfaction with friendship), and dispositional factors (e.g., personality traits).

The main predictors of life satisfaction are age, sex, income, education, and marital status. In other words, women and/or married people and/or those with high income always report higher levels of life satisfaction (e.g., Diener and Oishi, 2000; Diener and Biswas-Diener, 2002; Lucas et al., 2003; Stevenson and Wolfers, 2008; Cheung and Lucas, 2014; Grover and Helliwell, 2019; Joshanloo and Jovanović, 2020). However, there are some controversies regarding the association between age, education, and life satisfaction. Regarding the relationship between age and life satisfaction, some studies found that there was no association between age and life satisfaction (e.g., Diener and Suh, 1997), whereas others reported either a positive (e.g., Hansson et al., 2005) or negative association (e.g., Chen, 2001) between age and life satisfaction. Specifically, Helliwell and Putnam (2004) demonstrated that people aged above 65 had better life satisfaction compared to younger people. Moreover, according to one of the most impactful studies conducted in recent years regarding the two variables, life satisfaction has a U-shaped pattern that reaches its lowest point before increasing until later adulthood. Similarly, Löckenhoff and Carstensen (2004) found that subjective well-being either increases or is stable with age. Bartram (2021) found that there is a negligible post-middle-age increase in life satisfaction. One possible explanation for the controversy is that increases in some areas of life satisfaction offset the decreases in other areas as overall life satisfaction is made up of areas of life satisfaction (e.g., Malvaso and Kang, 2022). Regarding the associations between education and life satisfaction, some studies have identified positive associations between education and life satisfaction (Davis and Friedrich, 2004; Cheung and Chan, 2009), whereas others identified negative relationships between them (Rao et al., 2014). Moreover, studies have also found different mediators such as the mismatch between education and job (Artés et al., 2014) and educational aspirations that exceed opportunities (Ferrante, 2009) could mediate the negative effect of education on life satisfaction.

Friendship serves as a valuable source of social support across life spans, which provides psychological support for people who are facing stressful events (Brummett et al., 2005). Previous studies have found that the quantity of friends is associated with lower levels of depression in older adults (Potts, 1997). Moreover, support from friends has also been positively related to affective balance (Montpetit et al., 2017). Isolations from friends may be related to greater increases in psychological distress and depression compared to isolation from family (Taylor et al., 2018). Moreover, Van der Horst and Coffé (2012) have found that increasing the number of friends is associated with a reduction in stress. In addition, friendships in older adults have stronger associations with psychological well-being compared to family relationships and kin-based networks. Finally, the number of social relationships is related to the maintenance of low negative affect and high positive affect.

Moreover, friendship and social support are also closely related to life satisfaction (Kong et al., 2015; Tomini et al., 2016; Amati et al., 2018). Close friendships may result in high levels of well-being as indicated by self-esteem, psychosocial adjustment, and interpersonal sensitivity (Perry and Pescosolido, 2015). One study has found that participants with lifetime friendships are better adjusted compared to their peers (Gupta and Korte, 1994). Another study found that adults who have more satisfying and positive friendships have fewer feelings of hostility and anxiety (Bagwell et al., 2005). Moreover, social support tends to be positively associated with life satisfaction (e.g., Kong et al., 2015). One study looked at the number of Facebook friends in relation to life satisfaction and found a positive association between them (Bruine de Bruin et al., 2020).

There are also some predominant theories regarding the role of age in the association of social factors and life satisfaction such as the socioemotional selectivity theory (SST), which posits that people become increasingly conscious about how their time should be spent as they get older (Carstensen, 2006). This awareness makes them spend time on events that will make them as happy as possible (Carstensen, 2006). Thus, according to this theory, older adults would tend to have fewer friends compared to their younger counterparts as they will emphasize the emotional aspects of potential social interactions rather than the quantity of them.

Consequently, although there are some studies regarding the associations between the number of close friends and subjective well-being, none of them has tested the interrelation between the number of close friends and age in predicting life satisfaction. Hence, the aim of the current study is to test how the number of close friends is associated with life satisfaction. According to the prediction of the SST, the current study hypothesizes that the number of close friends should be most strongly related to life satisfaction in young people, less strongly related to life satisfaction in middle-aged adults, and weakly associated with life satisfaction in older adults as the emotional aspect of a friendship is the most important for older people but the quantity of close friends is not.

## Methods

### Data

This study used data from 29,785 participants with an age range from 16 to 101 years old from Wave 9 of the Understanding Society (University of Essex, 2022), which was collected between January 2017 and May 2019. All data collections have been approved by the University of Essex Ethics Committee. Participants completed informed consent before participating in these studies. Participants with any missing variables of interest were removed from further analyses.

### Measures

#### Number of close friends

The number of close friends was measured by the question “How many close friends do you have?,” which is a valid measure that has been used in a lot of studies (Latham-Mintus, 2019; Degro et al., 2021; Thompson et al., 2022).

#### Life satisfaction

Participants answered the question “How dissatisfied or satisfied are you with… your life overall?” using a 7-point scale ranging from 1 (not satisfied at all) to 7 (completely satisfied). The results of single-item measures and multi-item measures such as the Satisfaction with Life Scale (SWLS) have been shown to be very similar (Cheung and Lucas, 2014).

#### Control variables

Control variables included age, sex, monthly income, highest educational qualification, marital status, residence, and self-rated health (1 = very poor to 5 = excellent). How these variables were coded can be found in Table 1.

**Table 1:** Descriptive statistics of control variables, self-rated health, the number of close friends, and life satisfaction.

**Variables**	**Mean**	**S.D**.
Age	46.99	18.38
Monthly income	1,570.68	4,192.81
Self-rated health	3.50	1.09
Number of close friends	5.15	6.63
Life satisfaction	5.25	1.43
	N	%
**Sex**		
Male	13,300	44.65
Female	16,485	55.35
**Highest educational qualification**		
Below college	20,220	67.89
College	9,565	32.11
**Legal marital status**		
Single	14,510	48.72
Married	15,275	51.28
**Residence**		
Urban	22,703	76.22
Rural	7,082	23.78

### Analysis

For analysis, a hierarchical linear regression was used by taking predictors including age, sex, monthly income, highest educational qualification, marital status, residence, self-rated health, the number of close friends, and the interaction between age and the number of close friends (Aiken and West, 1991) to predict life satisfaction. As a *post-hoc* test to check the moderating role of age, three multiple regressions were used by taking predictors including age, sex, monthly income, highest educational qualification, marital status, residence, self-rated health, and the number of close friends to predict life satisfaction for young (below 35 years old), middle-aged (aged between 35 and 55 years old), and older people (above 55 years old) respectively, according to age cut-offs used by Petry (2002).

## Results

Descriptive statistics can be found in Table 1. The overall hierarchical regression model explained 8.7% of total variances in life satisfaction scores. The current study found that there is a significant interaction effect of age on the number of close friends (*b* = −0.003, *p* < 0.01, 95% C.I. [−0.004, −0.001]) after controlling for demographic covariates (Table 2). Simple slope regressions showed that the overall regression model explained 10.7, 13.3, and 11.1% of total variances in life satisfaction in young, middle-aged, and older adults respectively. Specifically, the number of close friends was positively related to life satisfaction in young people (*b* = 0.018, *p* < 0.001, 95% C.I. [0.012, 0.025]), less strongly associated with life satisfaction in middle-aged (*b* = 0.008, *p* < 0.001, 95% C.I. [0.004, 0.013]), and most weakly associated with life satisfaction in older adults (*b* = 0.004, *p* < 0.01, 95% C.I. [0.001, 0.007]; Table 3).

**Table 2:** The regression coefficient (*b*) for control variables, the number of close friends, self-rated health, and age by the number of close friend interactions with the total explained variances (R^∧^2).

**Variables**	***b***
Age	0.010^***^
Sex	0.75^***^
Monthly income	0.000^***^
Highest educational qualification	0.004^***^
Marital status	0.189^***^
Residence	0.084^***^
Self-rated health	0.411^***^
The number of close friends	0.024^***^
Age ^*^ The number of close friends	−0.003^***^
R^∧^2	0.087

* p < 0.05,

** p < 0.01,

*** p < 0.001.

All numbers were rounded up to three digits.

**Table 3:** The regression coefficient (*b*) for control variables, the number of close friends, self-rated health, and age by the number of close friend interactions with the total explained variances (R^∧^2) for each age group.

	***b***		
**Variables**	**Young**	**Middle-aged**	**Older**
Age	−0.021^***^	−0.002	0.027^***^
Sex	0.138^***^	0.076^**^	0.072^**^
Monthly income	0.000	0.000	0.000
Highest educational qualification	0.073^*^	0.093^***^	0.043
Marital status	0.346^***^	0.354^***^	0.290^***^
Residence	0.114^**^	0.082^**^	0.035
Self-rated health	0.428^***^	0.431^***^	0.3666^***^
The number of close friends	0.018^***^	0.008^***^	0.004^**^
R^∧^2	0.107	0.133	0.111

* p < 0.05,

** p < 0.01,

*** p < 0.001.

All numbers were rounded up to three digits.

## Discussion

The aim of the current study was to test how age may moderate the associations between the number of close friends and life satisfaction in a large sample of participants from the United Kingdom. Findings from the current study indicated that the number of close friends is generally positively related to life satisfaction. However, this association may depend on age. Specifically, the association between the number of close friends and life satisfaction was the strongest in young people, less strong in middle-aged, and the weakest in older adults.

Results of demographics in the model are consistent with findings that women and/or married people and/or individuals with a high income always report higher levels of life satisfaction (e.g., Diener and Oishi, 2000; Diener and Biswas-Diener, 2002; Lucas et al., 2003; Stevenson and Wolfers, 2008; Cheung and Lucas, 2014; Grover and Helliwell, 2019; Joshanloo and Jovanović, 2020). The positive association between age and life satisfaction was also consistent with previous studies (e.g., Hansson et al., 2005). The highest educational qualification was positively related to life satisfaction (Davis and Friedrich, 2004; Cheung and Chan, 2009). In addition, people who lived in the rural areas reported higher levels of life satisfaction. Finally, self-rated health was positively related to life satisfaction.

Findings from the current study were largely in line with the prediction of the SST, which proposed that quality rather than quantity of friendship matters in older age, which benefits well-being. Thus, the number of close friends may shrink with age and weakly connects with life satisfaction in older age. Moreover, these findings were still held after adjusting for self-rated health, which is strongly associated with objective health, (e.g., Wu et al., 2013). However, it is still possible that age differences in other unmeasured variables may play a role in the relationship between the number of close friends of life satisfaction. The SST proposes that older adults may intentionally choose their social networks to maximize their positive emotional experiences (English and Carstensen, 2014). Although the cross-sectional analyses in the current study could not shed light on the nature of the shifting of the focus from the number of friends to the quality of friendship, findings from other studies could provide support to this notion. For instance, the Berlin Aging Study explained the discontinuation of friendships in older adulthood as due to a lack of interest rather than an opportunity (Lang, 2000). In addition, Lansford et al. (1998) found that young people hope they have more friends but not old people. These findings make sense because older adults are unlikely to gain life satisfaction benefits by having more close friends.

### Implications of this study

Findings from the current study imply that interventions with the aim to improve well-being such as life satisfaction can be beneficial by helping recipients make more close friends. Such interventions may require different approaches in young and older people. Indeed, as pointed out by Bruine de Bruin and Bostrom (2013), effective interventions need a deeper understanding of the specific issues and what audiences need and want to be addressed. For instance, older adults may be more interested in maintaining their existing close friendships rather than building more close connections. Indeed, as pointed out by Fung et al. (2001), older adults may resist increasing their social networks through various activities as knowing new people may no longer be of importance to them (refer also Carstensen and Erickson, 1986; Korte and Gupta, 1991). Rather, older adults would prefer to reduce their feelings of loneliness through internet training (Choi et al., 2012), which may be explained by the notion that using these technologies can help them connect with the people that they most care about (Thayer and Ray, 2006; McAndrew and Jeong, 2012). On the contrary, younger people may be interested in growing their social networks and close friendships, but interventions can help them to avoid problems in their close friendships and prevent them from draining their emotional resources (Hartup and Stevens, 1999; Schlosnagle and Strough, 2017). Pro-social interventions can help younger people to grow their social networks and build close connections in a positive way.

### Limitations of this study and recommendations for future studies

Despite the strength of the current study including large sample size and well-controlled sociodemographic characteristics, self-rated health one limitation of the current study is its cross-sectional correlational nature, which cannot provide causality. Moreover, the current study relied on self-reported measures and did not have access to the actual social networks, it is certainly plausible that young people exaggerated their self-reported number of close friends and older adults underestimated theirs (Bruine de Bruin et al., 2020). Third, it is possible that unmeasured variables such as personality traits may influence the current findings. Moreover, the most obvious difference across age groups is health. Although the current study controlled for self-rated health, which is consistent with objective health (e.g., Wu et al., 2013) and declines with age (e.g., Zajacova et al., 2017), future studies should control for more objective health (e.g., physicians assessed health if possible). Finally, the question that asks about the number of close friends may not mean the same for different people. Further clarification is needed for future studies on this research topic.

## Conclusion

To conclude, the aim of the current study was to understand the association between the number of close friends and life satisfaction and test how this association may differ in different age groups. Findings revealed that there was a significant moderating effect of age on the association between the number of close friends and life satisfaction with the number of close friends most weakly related to life satisfaction in older adults. Despite some limitations, findings from the current study have important implications for interventions based on age that improve well-being in different age groups.

## Data availability statement

Publicly available datasets were analyzed in this study. This data can be found here: https://www.understandingsociety.ac.uk.

## Ethics statement

The studies involving human participants were reviewed and approved by University of Essex. Written informed consent to participate in this study was provided by the participants' legal guardian/next of kin.

## Author contributions

The author confirms being the sole contributor of this work and has approved it for publication.
